# Adenoma Formation following Limited Ablation of p120-Catenin in the Mouse Intestine

**DOI:** 10.1371/journal.pone.0019880

**Published:** 2011-05-17

**Authors:** Whitney G. Smalley-Freed, Andrey Efimov, Sarah P. Short, Peilin Jia, Zhongming Zhao, M. Kay Washington, Sylvie Robine, Robert J. Coffey, Albert B. Reynolds

**Affiliations:** 1 Department of Cancer Biology, Vanderbilt University, Nashville, Tennessee, United States of America; 2 Department of Bioinformatics, Vanderbilt University, Nashville, Tennessee, United States of America; 3 Department of Pathology, Vanderbilt University Medical Center, Nashville, Tennessee, United States of America; 4 Department of Morphogenesis and Intracellular Signaling, Institut Curie-Centre de National de la Recherche Scientifique, Paris, France; 5 Medical Service, VA Tennessee Valley Healthcare System, Nashville, Tennessee, United States of America; 6 Cell and Developmental Biology, Vanderbilt University, Nashville, Tennessee, United States of America; 7 Department of Medicine, Vanderbilt University Medical School, Nashville, Tennessee, United States of America; Northwestern University Feinberg School of Medicine, United States of America

## Abstract

p120 loss destabilizes E-cadherin and could therefore result in tumor and/or metastasis-promoting activities similar to those caused by E-cadherin downregulation. Previously, we reported that p120 is essential in the intestine for barrier function, epithelial homeostasis and survival. Conditional p120 ablation in the mouse intestine induced severe inflammatory bowel disease, but long-term cancer-related studies were impossible because none of the animals survived longer than 21 days. Here, we used a tamoxifen-inducible mouse model (Vil-Cre-ER^T2^;p120^fl/fl^) to limit the extent of p120 ablation and thereby enable long-term studies. Reducing p120 KO to ∼10% of the intestinal epithelium produced long-lived animals outwardly indistinguishable from controls. Effects of prolonged p120 absence were then evaluated at intervals spanning 2 to 18 months. At all time points, immunostaining revealed microdomains of p120-null epithelium interspersed with normal epithelium. Thus, stochastic p120 ablation is compatible with crypt progenitor cell function and permitted lifelong renewal of the p120-null cells. Consistent with previous observations, a barrier defect and frequent infiltration of neutrophils was observed, suggesting that focal p120 loss generates a microenvironment disposed to chronic inflammation. We report that 45% of these animals developed tumors within 18 months of tamoxifen induction. Interestingly, β-catenin was upregulated in the majority, but none of the tumors were p120 null. Although further work is required to directly establish mechanism, we conclude that limited p120 ablation can promote tumorigenesis by an indirect non-cell autonomous mechanism. Given that byproducts of inflammation are known to be highly mutagenic, we suggest that tumorigenesis in this model is ultimately driven by the lifelong inability to heal chronic wounds and the substantially increased rates of stochastic gene mutation in tissue microenvironments subjected to chronic inflammation. Indeed, although technical issues precluded direct identification of mutations, β-catenin upregulation in human colon cancer almost invariably reflects mutations in APC and/or β-catenin.

## Introduction

The inability to generate and maintain epithelial integrity is a major causative factor in inflammatory disease and cancer [1], [2]. In a number of inflammatory diseases, the initiating event is difficult to pinpoint because epithelial damage can induce inflammation and inflammation can induce epithelial damage. We showed previously that p120-catenin (hereafter p120) knockout in the epithelium of the mouse small and large intestines resulted in mucosal damage and inflammation leading to bleeding and death within the first three weeks of life [3]. The animals were born with a barrier defect suggesting that the inevitable inflammatory response was triggered by weak cell-cell adhesion and mucosal exposure to intestinal flora. Other mechanisms may contribute, however, as p120 knockdown in a polarizing colon cancer cell line (HCA-7) induces strong neutrophil attachment [3], and p120 ablation in the epidermis activates an NFκB-dependent inflammatory response but no barrier defect [4]. Mechanism aside, our previous mouse model precluded study of the possible tumorigenic effects of p120 loss due to the lethal consequences of knocking out p120 in a high proportion of the intestinal epithelium.

To date, much of the evidence linking p120 to human cancer is based on its physical and functional relationships with classical cadherins, E-cadherin in particular. Classical cadherins comprise a family of transmembrane cell-cell adhesion receptors important in development, morphogenesis, and cancer (reviewed in [5], [6]). They are regulated, in part, by p120-catenin and β-catenin, cytoplasmic armadillo repeat proteins that interact directly with distinct sites on the cadherin tail. β-catenin recruits α-catenin to the complex, and along with several other factors [7], [8], [9], [10] modulates functional interactions with the actin cytoskeleton [11], [12], [13]. In contrast, p120 binding controls cadherin stability and retention at the cell surface [14], [15]. If p120 is removed (e.g., by knockout or knockdown), classical cadherins, along with associated α- and β-catenins, are internalized and degraded. p120 also modulates the activities of several RhoGTPases [16], suggesting that the catenins in general coordinate a functional interface between cadherins and various other factors that interact with the actin cytoskeleton.

Epithelial (E)-cadherin is the most prominent cell-cell adhesion receptor in epithelial cells and is widely regarded as a master organizer of the epithelial phenotype [5]. It functions as a classic tumor suppressor in gastric cancer [17] and lobular carcinoma of the breast [18], where germline mutations in the E-cadherin gene are responsible for familial inheritance. In most other cancers, E-cadherin is considered a metastasis suppressor because it is downregulated in advanced tumors and plays a causal role in the transition to metastasis [19], [20], [21], [22], [23]. Because p120 regulates E-cadherin function and stability, it is widely suspected that p120 itself is a tumor and/or metastasis suppressor.

Over 85% of human colorectal cancer (CRC) begins with mutation of the *Adenomatous Polyposis Coli* gene (*APC*) [24]. APC loss-of-function stabilizes a pool of β-catenin, which then associates with the transcription factor TCF4 to drive transcription of the c-Myc oncogene and other cancer-relevant genes. Direct activation of β-catenin by mutation at regulatory serine/threonine phosphorylation sites has a similar effect, and, indeed, approximately 5% of human CRCs are thought to be initiated in this way [24]. Thus, mutations in *APC* and *β-catenin* are believed to account for the initiation of more than 90% of human CRC.

Notably, previous work suggests a cell-autonomous role for p120 in suppression of inflammation [4]. In contrast to the intestine, p120 ablation in the epidermis has no discernable effect on either cell-cell adhesion or barrier function despite reduced levels of both E- and P-cadherins. Instead, these mice develop severe epidermal inflammation due to cell-autonomous activation of NFκB [4]. Thus, in addition to its well-established role in cell-cell adhesion, p120 appears to play an important role in suppressing inflammation in keratinocytes, and probably other cell types.

Previously, we reported that p120 is essential in the intestine for epithelial homeostasis and life [3]. Using a Villin-Cre driver and our p120 conditional knockout mouse, we evaluated the effects of targeted p120 ablation in the small intestine and colon. Although superficially normal at birth, KO animals declined rapidly and died within 21 days. Cell-cell adhesion defects and inflammation led to progressive mucosal erosion and terminal bleeding. Interestingly, the mice were born with a chronic barrier defect, a condition almost always associated with inflammation. On the other hand, there was also evidence for other cell-autonomous effects of p120 downregulation. Specifically, neutrophil attachment *in vitro* to p120-deficient epithelial monolayers was dramatically increased and resulted in selective upregulation of COX-2 in the neutrophils. Thus, several different events associated with p120-deficiency appear to significantly promote inflammation.

Here, we report that p120 KO by itself, in just a small fraction of the intestinal epithelium (∼5–15%), results in a tumor incidence of 45% by 18 months post tamoxifen. Surprisingly, however, outright p120 loss was not observed in the tumor itself. Instead, p120 expression was retained in each of the 16 adenomas analyzed, albeit at apparently lower levels, indicating that p120 loss is not directly causative. The majority of adenomas, however, showed increased levels of cytoplasmic/nuclear β-catenin, suggesting driver mutations in APC, β-catenin, or other members of the canonical Wnt pathway. To approach mechanism, we examined *in vitro* effects of p120 KD in a colon cancer cell line (HCA-7) by gene expression and cytokine-array profiling, immunofluorescent staining and Real Time rtPCR. The striking upregulation of NFκB observed in p120 knockout epidermis was not evident [4], but cytokines associated with neutrophil infiltration (i.e., CXCL1, IL-8 and PDGF) were modestly elevated [25], [26], [27]. The latter is consistent with our observation of obvious neutrophil recruitment to p120 null areas. Collectively, these data raise the possiblility that the tumors result from an increased rate of gene mutation, a well-established consequence of long-term unresolved inflammation [28]. Over the lifetime of the animals, the cumulative effects of p120-associated inflammation are likely to be significant, and not unlike the tumorigenic effects of ulcerative colitis, where the risk of cancer is increased by ∼18% over 30 years [29].

## Results

### Regulated p120 KO in the small and large intestine

Previously, we reported that conditional p120 knockout in the small intestine and colon (using a constitutively expressed Villin-Cre mouse driver) causes a striking IBD-like condition and death within the first few weeks of life [3]. Here, we crossed the same conditional p120 KO mouse to a tamoxifen-inducible Villin-Cre transgenic mouse driver (Vil-Cre-ER^T2^) with the goal of limiting p120 KO to levels compatible with long-term survival. As with the previous model, the Vil-Cre-ER^T2^ mouse permits selective targeting of Cre-ER^T2^ expression to the epithelial compartment of the small and large intestines, but in addition provides the opportunity to precisely control the timing and amount of Cre activity through administration of tamoxifen [30].

**Figure 1:**
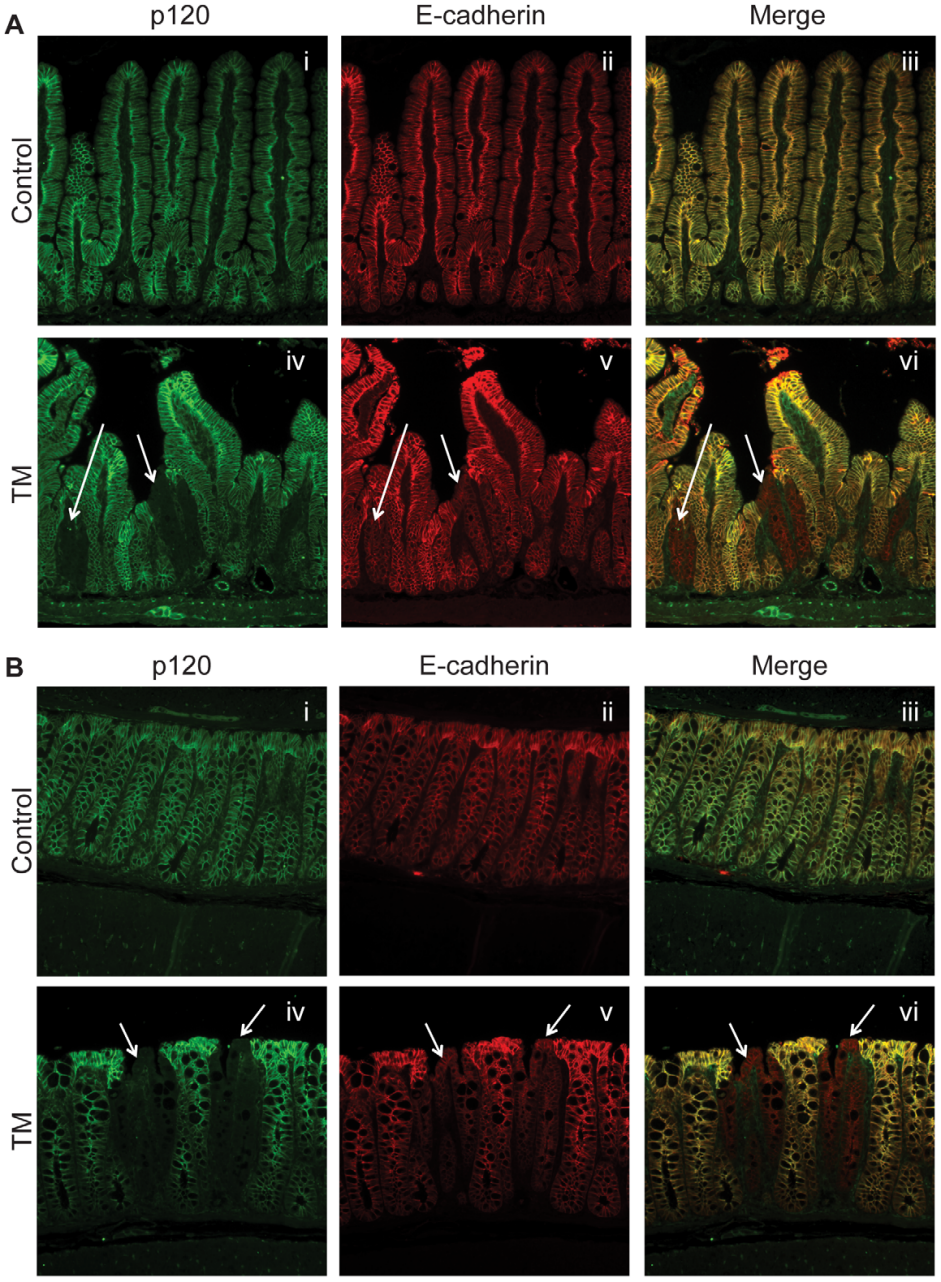
Limited ablation of p120 in the small and large Intestine. At the two-month time point sections of the small (A) and large (B) intestine were co-stained with p120 (i and iv) and E-cadherin (ii and v). Arrows indicate regions of p120 KO.

To identify an appropriate regimen of tamoxifen administration, the timing and dose of tamoxifen given was varied in a series of pilot experiments (not shown). The amount of p120 KO in the small and large intestines was visualized 2 months later by immunofluorescence staining of tissue sections (Figures 1A and 1B) and quantified (data not shown). Age-matched Vil-Cre-ER^T2^; p120^F/F^ treated with oil were used as controls for tamoxifen-treated animals to rule out the possibility of recombination in the absence of tamoxifen. The tamoxifen regimen selected (IP injection of 1 mg/20 g/day for 3 days) reproducibly induced ∼5% p120 knockout (1 out of every 20 crypts) in the small intestine (Figure 1A) and ∼15% knockout (3 out of every 20 crypts) in the colon (Figure 1B) with little, if any, overt effects on the health of the animals. p120 null crypts were usually distributed randomly throughout the small intestine and colon. The images in Figure 1 illustrate a cluster of p120-null crypts in order to show multiple examples of p120-negativity in a single figure. Interestingly, the levels of p120 ablation observed at two months persisted with little change over the lifetime of the animals (referred to hereafter as “limited” p120 KO). We were surprised to find that p120 ablation did not appreciably compromise progenitor cell function, and despite the effects of p120-loss on cell-cell adhesion, p120-null progenitor cells were properly retained within the stem cell niche. These experiments define an effective protocol for generating a consistent level of “limited” p120 KO for long term evaluation of the effects of p120 ablation in the intestine.

At two months post tamoxifen, p120 knockout in both the small and large intestines manifested as columns of p120-negative cells derived from the clonal progeny of p120-ablated stem cells. However, in the small intestine, p120 null villi were invariably broken off (arrows, Figure 1A, iv), apparently weakened by p120 loss and the resulting cell-autonomous downregulation of E-cadherin. This condition was chronic, apparently persisting for the lifetime of the animal. p120-null crypts remained in the small intestine, but p120-negative villi were rarely seen. In contrast, the p120 null columns in the colon were largely intact (Figure 1B, arrows), except for minor structural weakness and occasional signs of erosion at the top of the crypt (Figure 1B, arrows). The colon lacks the free-standing villi found in the small intestine, and instead is composed of deep crypts containing columns of epithelial cells that physically support one another. Because of this arrangement, pockets of p120-null tissue are surrounded by columns of wild type tissue that appear to protect the p120-null columns and thereby mask the effects of structural weakness.

### Limited p120 KO induces formation of cystic crypts

**Figure 2:**
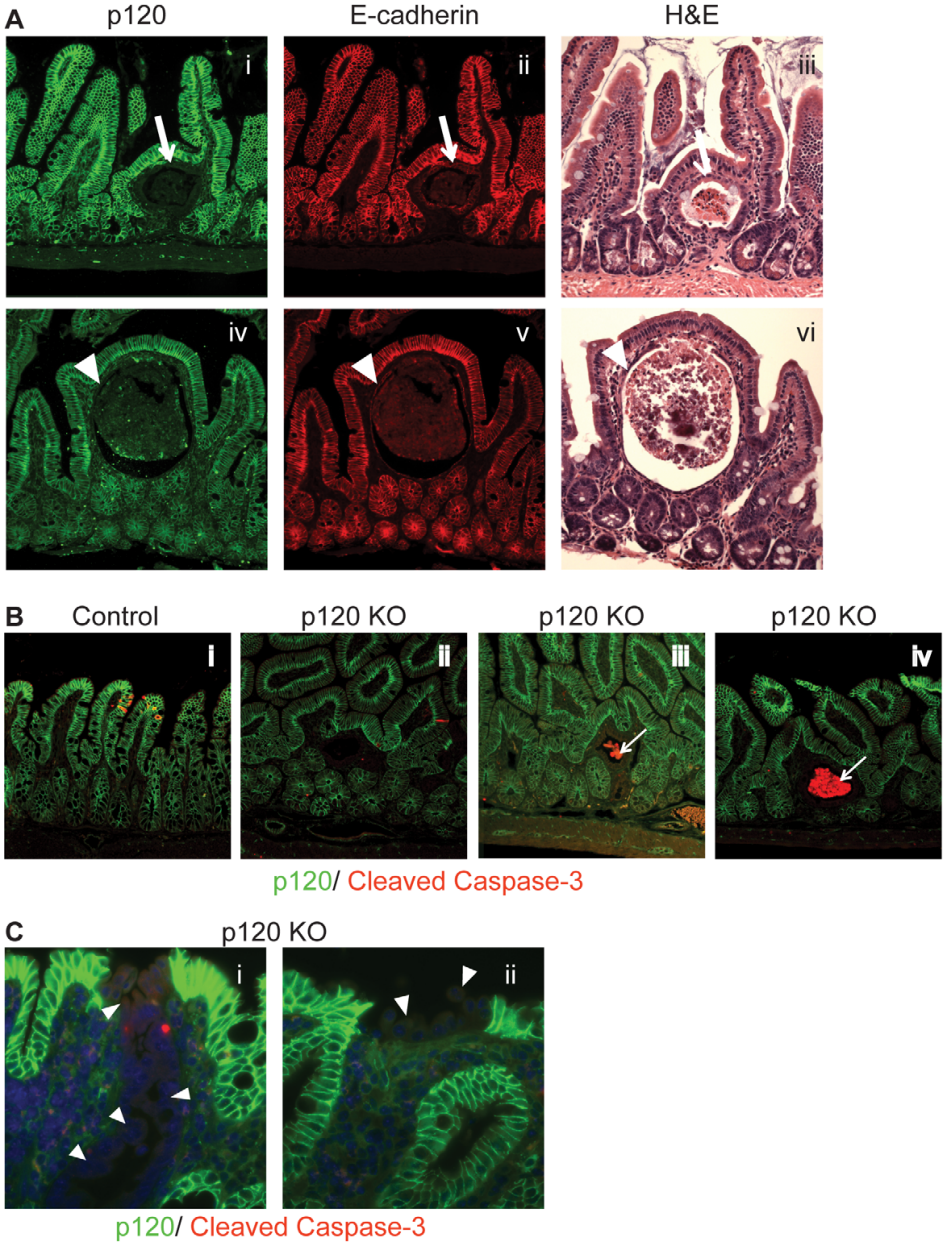
Cystic crypts develop in the absence of p120. At the two-month time point small intestine sections were (A) co-stained with p120 and E-cadherin or hematoxylin and eosin. Arrows and arrowheads indicate the p120-negative epithelium lining the cyst walls. 20× magnification. (B) and (C) WT and p120 KO small intestine tissue was co-stained for p120 and cleaved caspase-3, a marker for apoptosis. Eleven of the twelve cysts stained contained apoptotic cells (arrows). 20× magnification. (C) Arrowheads point to poorly adhesive, rounded cells which often result from p120 loss. 63× magnification.

To further examine the effects of limited p120 ablation over time, tamoxifen-treated animals were aged and then sacrificed at 2, 4, 6, 8, 12, and 18 months post tamoxifen. Paraffin-embedded sections were initially examined by hematoxylin and eosin (H&E) staining and by double immunofluorescent staining with antibodies to p120 and E-cadherin (Figure 2A) to evaluate the effects of p120 ablation at the tissue level. Interestingly, a small number of cystic crypts were observed in p120 KO, but not control animals. Quantification revealed an average of ∼5 cystic crypts per p120 KO animal, all of which were derived from p120 null crypts. The size of the cysts in any given animal varied widely (e.g., Figure 2A, compare top and bottom panels) and this was true at every time point from 2 to 18 months. The average number of cysts per animal was relatively constant over this period, suggesting a steady state condition whereby new cysts emerge on a regular basis and large cysts are lost and/or removed. Notably, the height of the epithelial lining of the smaller cysts (Figure 2A, arrows) is relatively normal but becomes progressively thinner as the cysts enlarge. For example, the lower panels (Figure 2A) show an enlarged cyst encased by an epithelial lining that has become so thin as to be almost unrecognizable (arrowheads). Overall, these data suggest that p120-null crypts transform at a low but steady rate into cysts, which expand in a predictable fashion until they are eventually reabsorbed or ruptured.

The cysts arise initially from p120 null crypts that are indistinguishable from others until they begin to increase in size. Presumably, the stem cells that normally reside in the crypts are viable and continue to generate new cells that are shed into the lumen and die. To evaluate the fate of these cells, a panel of 12 small to moderately sized cysts was analyzed by staining for cleaved caspase-3, a marker of apoptosis (Figure 2B). Of these, one appeared to lack apoptotic cells (panel ii) whereas the other 11 ranged from a few (panel iii) to many (panel iv) apoptotic cells, suggesting that the most prominent contents of late-stage cysts are remnants of apoptotic epithelial cells. In general, neutrophils were also observed in and around p120-negative cysts and cyst contents appeared to evolve along with increasing size into a more complicated mixture of apoptotic, necrotic, and inflammatory cells.

Figure 2C illustrates a rare example in the small intestine where adjacent columns of p120-null cells appeared to protect an entire villus wall, permitting its extension to the surface of the lumen (panel i). Panel ii illustrates adhesive failure of unprotected p120-null cells relative to surrounding p120 intact counterparts.

### p120 ablation in the intestine does not by itself increase cell proliferation

**Figure 3:**
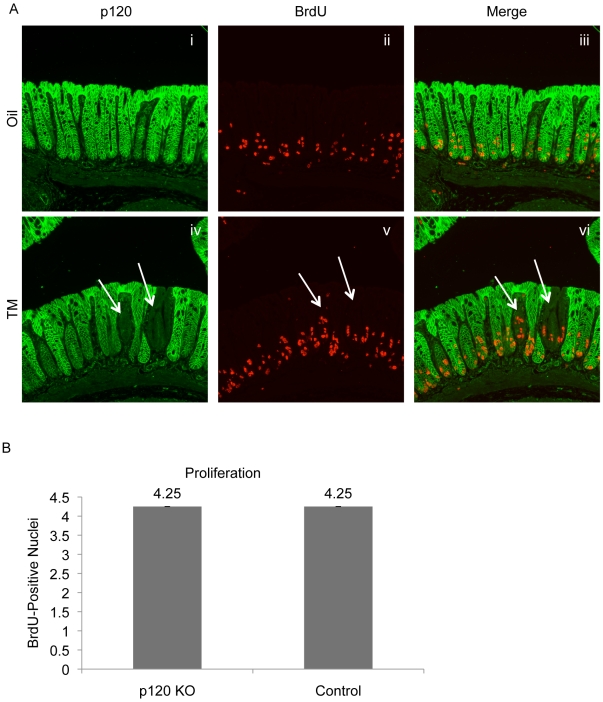
Proliferation is normal in the limited p120 ablation intestine. At the two-month time point, mice were injected with BrdU and sacrificed 2 hours later to measure proliferation. (A) Colon sections were co-stained for p120 and BrdU. Arrows indicate regions of p120 KO. (B) BrdU-positive nuclei were quantified in control and p120 KO tissue. No significant difference in proliferation was observed (p = 1) as both control and knockout tissues had an average of 4.25 BrdU-positive nuclei/ crypt.

Previously, we observed that there was a uniform 3-fold increase in intestinal epithelial proliferation in the constitutive p120 KO mouse [3], but it was not possible to distinguish between cell-autonomous effects versus a spectrum of significant indirect effects associated with tissue damage and widespread inflammation. Therefore, we revisited the issue here using the Vil-Cre-ER^T2^/p120 KO mice where cell-autonomous effects of p120 ablation were largely confined to small pockets of p120 ablation (e.g., Figure 3, arrows). Under these conditions, BrdU pulse-chase experiments conducted 2 months post tamoxifen show clearly that p120 ablation by itself has very little effect on proliferation (Figure 3). p120-null crypts were identified by immunofluorescence and selectively analyzed for BrdU-positive cells (Figure 3A, arrows). The number of BrdU-positive nuclei/ crypt was compared to that of control animals lacking p120 knockout. No significant difference in proliferation was observed (p = 1) as both showed an average of 4.25 BrdU-positive nuclei/ crypt (Figure 3B).

### Neutrophil infiltration and barrier dysfunction are directly associated with p120 ablation

**Figure 4:**
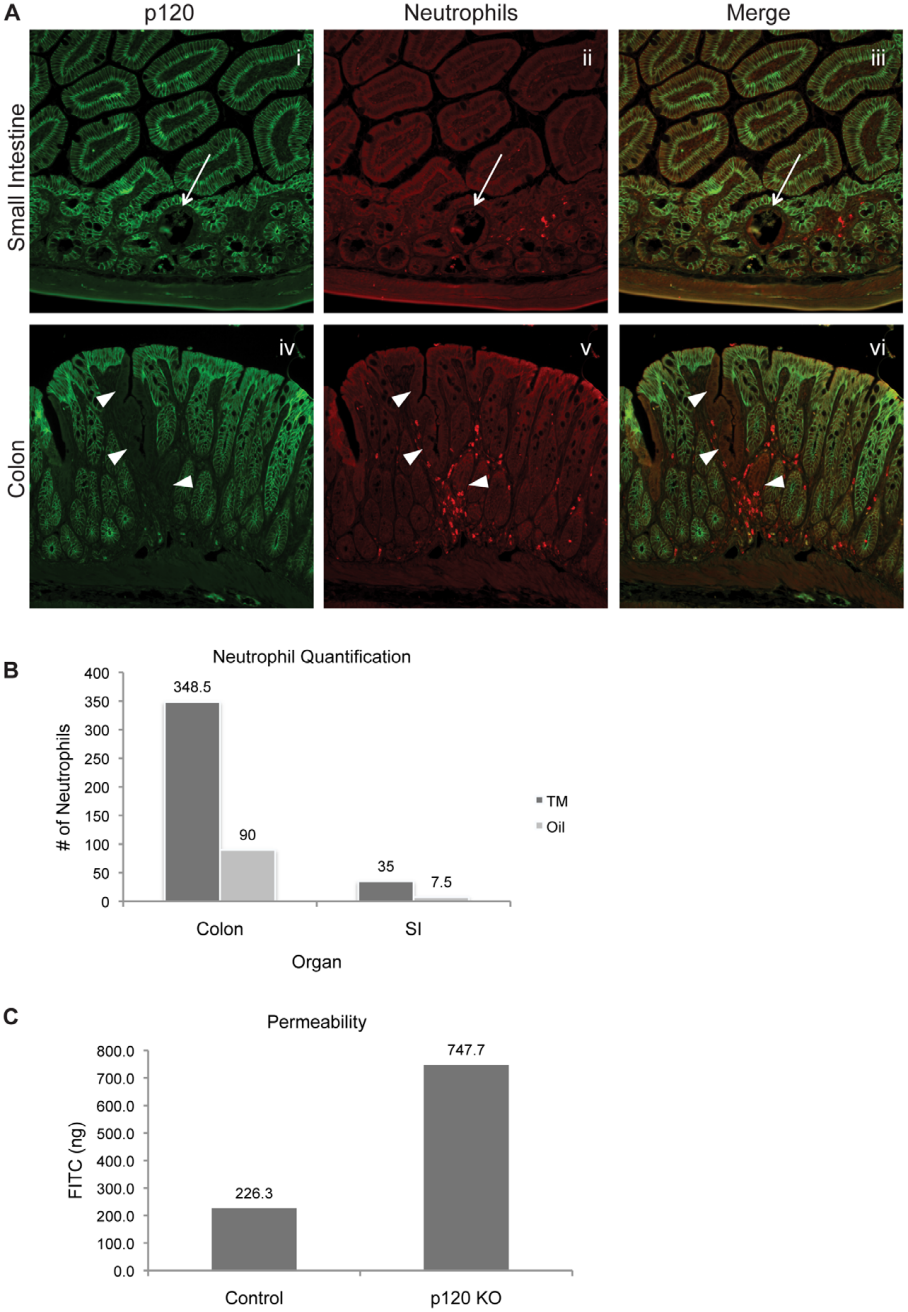
Neutrophils infiltrate p120 null regions of the small intestine and colon. (A) p120 KO small intestine and colon sections were co-stained for p120 and neutrophils. Arrows and arrowheads indicate regions of p120 ablation. (B) Neutrophils were quantified in oil and tamoxifen-induced small intestine (p = .06) and colon (p = .0092) samples. (C) At the two-month time point, mice were gavaged with FITC-dextran, and serum levels of FITC-dextran were measured to test for an intestinal permeability defect. FITC-dextran levels were significantly higher (3.3-fold) in p120 KO mice (p = .03).

We observed previously in our constitutive p120 KO mouse that epithelial proliferation was uniformly increased due to a widespread inflammatory field effect [3]. However, *in vitro* experiments with HCA-7 KD cells revealed a striking cell-autonomous increase in neutrophil attachment to p120-deficient monolayers. Here, although the effects of p120 ablation on cell proliferation (relative to the previous model) were reversed by limiting the extent of p120 KO, selective recruitment of neutrophils to p120-null fields was retained (Figure 4A). Recruitment was more pronounced in the colon, but was also observed clearly in the small intestine, as quantified in Figure 4B. Neutrophil numbers in the p120 KO colon were 3.9-fold higher than in control colons (p = .0092). The average number of neutrophils in the p120 KO small intestine was 4.7-fold higher than in control small intestine tissue, but the result narrowly missed statistical significance (p = .06). These observations suggest that p120 ablation affects the immediate microenvironment in ways that participate in both the homing and attachment of neutrophils to pockets of p120 null epithelium.

In addition, *in vivo* permeability assays revealed a clear barrier defect (Figure 4C), even though the p120 KO component of the overall gut lining was on average less than 10% of the total epithelium. p120 KO and control animals were gavaged with FITC-dextran and assayed 4 h later for leakage of FITC-dextran into the bloodstream. Relative to control mice, serum levels of FITC-dextran were 3.3-fold higher in Vil-Cre-ER^T2^/p120 KO mice (p = .03), indicating that even limited p120-ablation (5–15%) leads to readily detectable defects in epithelial permeability.

### Limited p120 ablation indirectly induces tumorigenesis

**Figure 5:**
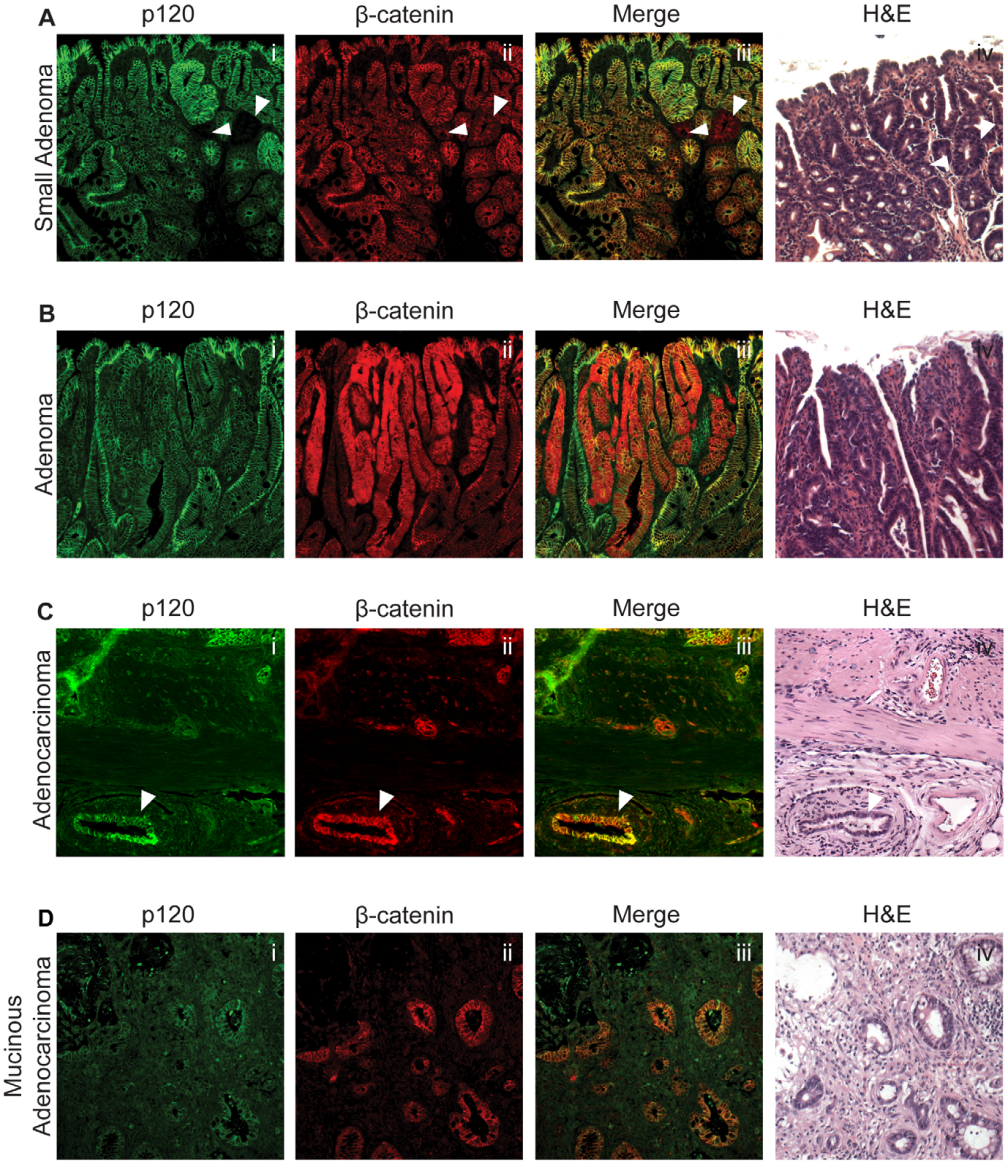
p120 is decreased, not gone, in tumors from p120 KO mice. p120 KO tumors of different stages were co-stained with p120 and β-catenin or H&E. (A) Small adenoma. Arrowheads indicate p120-null crypts. (B) Adenoma (C) Adenocarcinoma. Arrowheads point to invasive adenocarcinoma. (D) Mucinous adenocarcinoma. All of the tumors examined exhibited reduced but not complete loss of p120.

Although several lines of evidence have suggested a tumor suppressor role for p120, we did not expect that p120 ablation in the absence of other genetic defects would be tumorigenic. Surprisingly, intestinal tumors were detected in about half of the mice at the 12-month time point and then again at 18 months. Table 1 combines the tumor data from both time points and breaks it down according to tumor type, location, number, and expression patterns of β-catenin. Overall, 45% (14/31) of the Vil-Cre-ER^T2^/p120 KO mice examined at 12 or 18 months developed tumors while none were detected in age matched control (n = 29) animals. The tumors occurred in the small intestine or the cecum and were classified as one of four types by expert pathologist, Dr. M.K. Washington: small adenoma (Figure 5A), adenoma (Figure 5B), adenocarcinoma (Figure 5C), and mucinous adenocarcinoma (Figure 5D). The adenocarcinoma and mucinous adenocarcinomas were locally invasive, but invasion into the vasculature was not observed. Liver tissue was collected from mice at both 12- and 18-month time points, but metastases were not visible grossly or with H&E staining.

**Table 1:** Tumor types and histological patterns.

Tumor type	Age	Location	# of Tumors	β-catenin
Small Adenoma	12 months	Small Intestine	3	Junctional
Small Adenoma	12 months	Small Intestine	1	Cytoplasmic/nuclear
Small Adenoma	18 months	Cecum	1	Junctional/cytoplasmic
Adenoma	12 months	Small intestine	3	Cytoplasmic/nuclear
Adenoma	18 months	Small Intestine	4	Cytoplasmic/nuclear
Adenocarcinoma	18 months	Jejunum	1	Cytoplasmic/nuclear
Mucinous Adenocarcinoma	18 months	Ileum	1	Junctional
Mucinous Adenocarcinoma	12 months	Cecum	2	Junctional
**Total number of tumors**			**16**	**10/16**

Given the relatively high tumor incidence, we then examined p120 levels by immunofluorescent staining expecting to find that most, if not all tumors would be p120 null. Instead, none of the 16 tumors lacked p120, although in most cases p120 staining appeared to be selectively reduced. On extremely rare occasions, a few p120-negative crypts were found alongside adenomatous tissue (e.g., Figure 5A, arrowheads), but we were not able to find any examples of p120 loss that were obviously adenomatous. Thus, the mechanism of tumorigenesis is mostly likely indirect.

Nonetheless, p120 levels were consistently reduced in both small and midsized adenomas (e.g., Figures 5A, i; 5B, i). The top panels (i and ii) in Figures's 5A and 5B were double stained with antibodies to p120 and β-catenin, respectively. Interestingly, in the small adenomas, β-catenin staining was unremarkable and essentially identical to that of p120 in both location and intensity. In striking contrast, the large adenomas were defined by marked upregulation and mislocalization of β-catenin (Figure 5B, ii), whereas p120 levels remained decreased (Figure 5B, i), as also observed in the small adenomas (Figure 5A, i).

Three of the sixteen tumors were classified as mucinous adenocarcinomas (Figure 5A, ix–x), which are thought to arise through loss of TGFβ signaling [31], [32]. Similar tumors have been observed in Smad3 mutant mice [32], [33]. The presence of tumors arising through different (i.e., Wnt- and TGFβ-associated) pathways is consistent with a primary mechanism associated with increased overall rates of gene mutation.

### p120 loss induces expression of neutrophil chemoattractants

**Figure 6:**
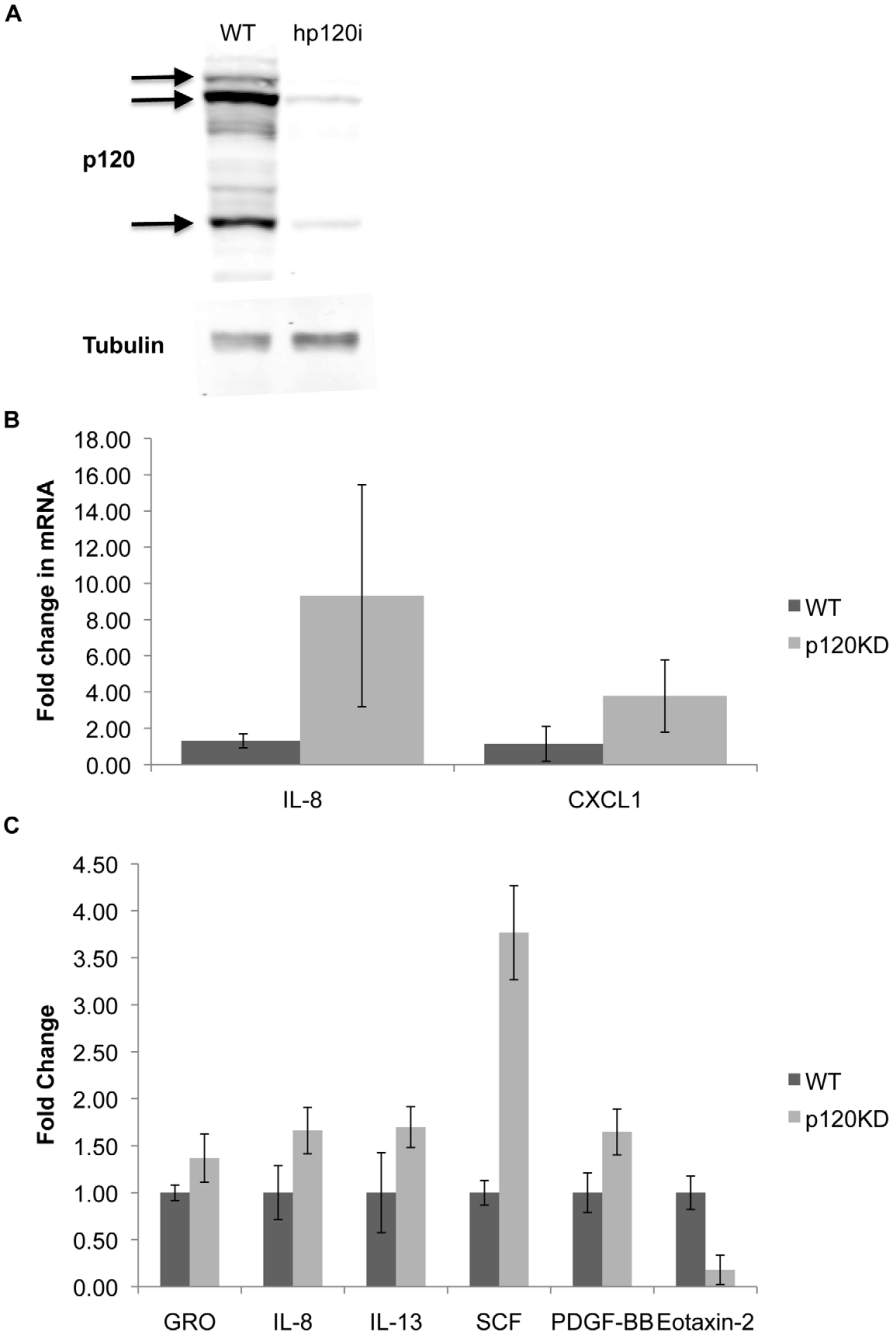
Cytokines are upregulated in p120 KD HCA-7 cells. (A) Western blot for p120. Control HCA-7 cells are in lane 1 and p120 KD cells are in lane 2. Tubulin is shown as a loading control (B) Real time rtPCR for IL-8 and CXCL1. IL-8 and CXCL1 levels were significantly higher (p = .013 and p = .015 respectively) in p120KD HCA-7 cells than WT HCA-7 cells. (C) Cytokine array on proteins secreted into cell media. GRO (p = .06), IL-8 (p = .0036), IL-13 (p = .012), SCF (p = .012) and PDGF (p = .039) were increased, and Eotaxin-2 (p = .03) was decreased in p120KD HCA-7 cells. Data = mean ± standard deviation.

Studies of p120 knockout in the skin suggest that p120 ablation can induce inflammation by cell-autonomous mechanism(s) independent of barrier status [4]. Whether a similar barrier-independent mechanism exists in the intestine is unknown. To examine effects of p120 knockdown in an environment where the barrier defect is unlikely to be relevant, we performed preliminary microarray experiments in the p120 KD HCA-7 cell system described previously [3]. First, to examine the level of p120 KD, Western blots were performed (Figure 6A). Subsequent preliminary microarray experiments indicated that several key mediators of neutrophil recruitment were modestly upregulated in the absence of p120 (e.g., IL-8, CXCl1, PDGF-BB), providing a partial explanation for the recruitment of neutrophils [25], [26], [27].

CXCL1 and IL-8 were further validated by Real Time rtPCR(Figure 6B). For CXCL1 (GRO), IL-8 and PDGF-BB, secreted protein levels were quantified directly by cytokine array using media supernatants (Figure 6C). Collectively, the data is consistent with the notion that p120-deficiency upregulates genes involved in neutrophil attraction [25], [26], [27], as well as the proinflammatory cytokines IL-13 and SCF (Figure 6C). The former is consistent with selective homing of neutrophils to pockets of p120-null epithelium, while the latter supports prior findings in skin suggesting that p120 suppresses inflammation.

## Discussion

We showed previously that constitutive p120 KO in the mouse intestine induces severe and ultimately lethal inflammatory bowel disease [3]. However, we were unable to evaluate effects of p120 ablation on tumorigenesis because the animals died by 21 days of age. Here, we have used an inducible system to limit the extent of p120 knockout to levels that do not cause IBD or other overt abnormalities. We report that under these conditions, almost half of the animals develop adenomas with a latency of 12–18 months.

Taken together, the results are highly reminiscent of the DN-cadherin mouse model of inflammatory bowel disease, in which mice develop a Crohn's-like phenotype by three months of age and adenomas by six [34]. We (and others) have shown previously that DN-cadherin expression causes internalization and degradation of endogenous cadherins by sequestering p120 [3], [14], [15], [35]. Thus, p120-ablation and forced expression of DN-cadherin appear to be mechanistically equivalent and have the same spectrum of short and long-term consequences in the intestine. These observations suggest a physiologically relevant context for interpretation of DN-cadherin phenotypes. Specifically, DN-cadherin phenotypes in mice most likely predict consequences of p120 downregulation, a phenomenon frequently observed in human epithelial cancers [36].

Unexpectedly, although microdomains of p120 KO were ubiquitously present throughout the intestinal epithelium, they were virtually absent in adenomas arising from these animals. It is increasingly evident, however, from our unpublished work in various APC mutant mouse models that outright p120 ablation (as opposed to reduced p120 levels) may be incompatible with cell viability in APC-mutant adenomas (data not shown). Together, these observations indicate that the tumorigenic mechanism associated with limited p120 ablation is not cell autonomous, but instead derives from secondary effects of p120 loss that influence cell-transformation in nearby p120 positive cells. The simplest explanation is that foci of p120-null tissue comprise chronically inflamed microenvironments that persist throughout the intestine for the lifetime of the animal. Chronic inflammation is a well-established driver of tumorigenesis [37] associated with toxic byproducts (e.g., reactive oxygen species [28], [38]) and high DNA mutation rates [37], [39]. Although technical and tissue limitations precluded efforts to identify specific gene mutations, β-catenin upregulation was used as a surrogate marker to show clear canonical Wnt pathway activation in 10 of 16 tumors analyzed (e.g., Figure 5B). Collectively, these findings are consistent with a tumorigenic mechanism involving chronic inflammation and gene mutations in APC, β-catenin or other members of the canonical Wnt pathway [40], [41], [42].

Notably, ulcerative colitis (UC) patients have an estimated 18% risk of developing CRC within 30 years of disease onset, and approximately 15% of patients with inflammatory bowel disease die of CRC [29]. Several relevant mouse models have been developed to study colitis-associated cancer (CAC). For example, IL-10 knockout mice exhibit a generalized enterocolitis and, in fact, 60% develop colorectal adenocarcinomas by 6 months of age [43]. Furthermore, in the dextran sulfate sodium (DSS) model of chronic colitis, 50% of mice develop tumors within 2 weeks following the last of nine DSS cycles [44], an incidence almost identical to that of our model. DSS alone is not an efficient model for studying CAC, so the more commonly utilized model employs a single dose of azoxymethane to initiate tumorigenesis followed by 3 cycles of DSS [45]. Tumors are observed throughout the colon with the highest concentration occurring in the left colon where the inflammation is most severe [45]. Although an exogenous mutagen was not used in our model, the overall process is probably similar in that both are likely to be driven by inflammation and ultimately mutation of known colon cancer genes (e.g., members of Wnt and/or TGFβ pathways).

Since the tumors in our model retained low levels of p120 expression, we explored the possibility that p120 is a haploinsufficient tumor suppressor. Tamoxifen-treated Vil-Cre-ER^T2^;p120^F/+^ mice were aged for 15 months (data not shown). No difference between heterozygous and wildtype mice was observed suggesting that haplosinsufficiency is not responsible for tumorigenesis in Vil-Cre-ER^T2^/ p120 KO mice.

A central goal of this study was to reduce the extent of p120 knockout in theintestinal epithelium to levels that permitted long-term analyses. Indeed, with knockout levels of ∼10%, the animals lived to old age and were outwardly identical to control littermates. The widespread inflammatory field effect observed in the previous model (following constitutive p120 KO) resolved completely, as did elevated rates of apparent compensatory cell proliferation. In contrast, the barrier defect did not resolve, confirming that reduced barrier function is indeed a cell-autonomous effect of p120-loss. Another direct consequence of p120 ablation was observed primarily in the small intestine, where p120-negative villi were invariably damaged or broken off altogether (Figure 1A), apparently reflecting persistent irreparable wounds (e.g., Figure 2C). In the colon, p120-negative crypts remained largely intact because the colonic architecture physically protected them (Figure 1B). The relatively selective effect of p120 ablation on the small intestine architecture could account, in part, for the higher tumor incidence in the small verses large intestine. For example, in humans with colitis-associated cancer, neoplastic regions are generally located adjacent to regenerative mucosa [45] which may undergo similar cycles of chronic wounding and repair. Lastly, the consistent appearance of cystic crypts in our mice was clearly dependent on p120 knockout, but the cause(s) and/or consequence(s) of cystic crypt formation are unknown. They clearly were not direct tumor precursors because none of the tumors from these mice were p120 null, but the cysts were often associated with neutrophilic infiltrates and inflammation. Though not necessarily related, cysts are frequently observed in human CRC and in adenomas in most mouse models of colon cancer [43], [46], [47].

Although obvious neutrophil homing to p120 negative foci was observed, we were largely unsuccessful in attempts to definitively link p120 ablation to particular cell autonomous mechanisms of neutrophil recruitment. Neutrophils have positive roles in tissue repair [48], but also generate reactive oxygen and nitrogen species [28], [38], which can lead to DNA damage and tumorigenesis [37], [39]. Micro- and cytokine-array experiments did turn up modestly elevated mRNA and protein expression levels of neutrophil-attracting chemokines, CXCL1 and IL-8, and other potentially relevant factors (Figure 6), following *in vitro* knockdown of p120 in HCA7 cells. However, the effects overall were moderate and appeared insignificant relative to the extraordinary upregulation of cytokines associated with p120 ablation in skin.

Overall, the data reveal that stochastic p120 knockout in the mouse intestine can lead to adenoma formation, consistent with the possibility of a tumor suppressor function. Although these experiments were, in fact, designed to evaluate this possibility, our original working hypothesis was based conceptually on well-established effects of E-cadherin downregulation in cancer (i.e., increased cell motility, epithelial-mesenchymal transition, invasiveness and/or reduced contact inhibition of cell growth). Clearly, these would be cell autonomous effects of p120 loss and are therefore inconsistent with the fact that none of the tumors from these animals were p120 null. Instead, our results suggest a more complicated scenario that may in part reflect a key difference between tumor initiation and subsequent events relevant to tumor progression. Tumorigenesis in these animals is most likely a secondary effect of lifelong chronic inflammation, excessive DNA damage, and ultimately, direct mutation of genes already known to promote intestinal cancer. Whether p120 downregulation does in fact constitute a causal factor in human IBD and/or intestinal cancer remains unclear. However, the frequent occurrence of p120 downregulation in most epithelial cancers, including CRC [49], [50], [51], is extensively documented (reviewed in [36]), and important roles for p120 downregulation in human IBD and/or cancer remain an interesting possibility.

## Materials and Methods

### Ethics Statement

All experiments involving animals were approved by the Vanderbilt University Institutional Animal Care and Use Committee for protocol numbers M/05/116 and M/07/061.

### Mice

Mice were maintained under a strict 12-hour light/dark cycle and with free access to chow and water. Mice containing the floxed p120 allele (F) were generated as described previously by our lab [52]. p120^F/F^ mice (fully backcrossed onto a C57Bl/6 background) were crossed with Vil-Cre-ER^T2^ mice [30] to target inducible p120 ablation to the small intestine and colon. Six-week-old Vil-Cre-ER^T2^; p120^F/F^ were intraperitoneally injected with 1 mg/20 g/day of tamoxifen for 3 days. Age-matched Vil-Cre-ER^T2^; p120^F/F^ treated with oil were used as controls for tamoxifen-treated animals.

### Immunohistochemistry

Swiss-rolled intestinal tissue was fixed in formalin overnight at 4°C and then embedded in paraffin. Paraffin-embedded sections (5 µm) were prepared for H&E staining and immunohistochemistry (IHC). Sodium citrate antigen retrieval was used for all antibodies except for anti-BrdU. For fluorescent IHC, samples were incubated with primary and secondary antibodies overnight at 4°C and for 2 hr at room temperature, respectively.

The following primary antibodies were utilized: rabbit anti-p120 (F1αSH); pp120 (#610133) and anti-E-cadherin (#610181) from BD Transduction Laboratories; anti-β-catenin (#C2206 Sigma); anti-BrdU (Accurate Chemical & Scientific Corp. #OBT0030); rat-anti-mouse neutrophils (#MCA771GA Serotec); and anti-cleaved caspase-3 (Cell Signaling). Secondary antibodies, conjugated to either the 488 Alexa-fluor or the 594 Alexa-fluor, were obtained from Molecular Probes.

### Proliferation

To determine proliferation rates, mice were injected intraperitoneally with BrdU 2 h before sacrificing. BrdU-positive nuclei were counted in p120-null crypts and compared to the number in oil-treated control mice.

### *In vivo* permeability assay

Intestinal paracellular permeability was determined by measuring the appearance of a marker, FITC-dextran (4.4 kDa FITC-dextran; Sigma), in blood [53]. Briefly, control and p120 KO mice were fasted for 12 hours. Then, 22 ml/kg body weight of PBS (pH 7.4) containing 22 mg/ml FITC-dextran were administered by gavage. Four hours later a blood sample (150 µl) was obtained and centrifuged to separate plasma. Plasma (50 µl) was mixed with an equal volume of PBS (pH 7.4). The concentration of fluorescein was determined using a fluorimeter with an excitation wavelength at 485 nm and an emission wavelength of 530 nm using serially diluted samples of the marker as standard.

### Cell lines, reagents, constructs

Cell culture conditions for HCA-7 cells have been described previously in detail [3]. Lentiviral vectors containing a GFP cassette and expressing either shRNA directed against human p120 or an empty control vector were constructed in pLentiLox (pLL) 5.0 vectors [54]. Production of pLL virus for shRNA expression was conducted in HEK293T cells as described [54].

### Quantitative PCR and Cytokine array

HCA-7 cells with and without p120 were plated on transwell filters (pore size 0.3 microns) and cultured for 10 days as described previously [3]. RNAs were then isolated and quantitated by Real Time rtPCR [4] and repeated three times.

For cytokine arrays, HCA-7 cells were cultured as above. Regular media was replaced with serum free media and incubated for 24 hrs. Conditioned media from top and bottom chambers were mixed and applied to Human Cytokine Array 5 (RayBio) and results measured. The positive control value was used to normalize results from three different experiments. For each cytokine, the signal from control serum free media was subtracted from WT and p120KD conditioned media signals. Fold difference was calculated for each pair of WT/p120KD conditioned media signals and the average and standard deviation were calculated.

### Statistics

To determine statistical significance, the Student's t-test and ANOVA were used. A p-value of <0.05 was considered statistically significant.
